# Plasma-Sprayed Bioactive Ceramic Coatings with High Resorption Resistance Based on Transition Metal-Substituted Calcium Hexaorthophosphates

**DOI:** 10.3390/ma12132059

**Published:** 2019-06-27

**Authors:** Robert B. Heimann

**Affiliations:** Am Stadtpark 2A, D-02826 Goerlitz, Germany; robert.heimann@ocean-gate.de

**Keywords:** NaSiCons, NZP structure, osseoconductive coatings, plasma spraying, solubility, ionic conductivity, cell proliferation, *in vivo* testing

## Abstract

Calcium (titanium, zirconium) hexaorthophosphates with a [NZP] (sodium zirconium phosphate) structure belonging to the NaSiCon (**Na** **S**uper**i**onic **Con**ductor) family were deposited by atmospheric plasma spraying onto the surfaces of Ti6Al4V substrates. (NaSiCon *sensu strictu* refers to solids with the chemical formula Na_1+x_Zr_2_Si_x_P_3−x_O_12_, 0 < x < 3. In a broader sense, it is also used for similar compounds where Na, Zr and/or Si are replaced by isovalent elements). The microstructure of the coatings revealed the incongruent melting of the precursor material as ascertained by electron probe microanalysis (EPMA). The adhesion of the coatings to the substrate surface was within the limits specified for biomedical coatings. The solubility of the coatings was tested by immersion in 0.2 molar tris–hydroxymethyl–amino–methane–HCl (TRIS–HCl) buffer and found to be at least one order of magnitude lower than that of conventional hydroxylapatite coatings deposited under comparable conditions. *In vitro* biocompatibility tests with primary rat bone marrow cells (BMCs) showed a substantial cell proliferation in the presence of fetal bovine serum. Animal tests confirmed that coatings based on calcium (titanium, zirconium) hexaorthophosphates applied to Ti6Al4V rods implanted in the femoral medulla of sheep led to the strong neoformation of dense bone at a stable interface implant-bioceramic coating without coating delamination. Hence, based on their multifarious advantageous properties in the biomedical context, CaTi_4-x_Zr_x_(PO_4_)_6_ ceramics may be considered the ‘Sleeping Beauty’ of osseoconductive coatings for the stem of hip endoprostheses and dental root implants, osteosynthetic fixation devices, and bioelectric devices including bone growth stimulators.

## 1. Introduction

Calcium phosphates, including hydroxylapatite, are copiously used for biomedical applications ranging from coating the stem of hip endoprostheses, densified monolithic implants for dental root replacements, material for filling and repairing bone cavities, to bone growth-supporting 3D-scaffolds. This broad field of biomedical application of hydroxylapatite is grounded on its chemical and structural similarity to biological, i.e., calcium-deficient, defect apatite present in bone. Osseoconductive, i.e., bone-growth supporting, hydroxylapatite coatings are commonly applied by thermal, most often plasma spray, technology.

However, although hydroxylapatite is the acknowledged bioceramic workhorse in many biomedical applications, for some uses the limited chemical stability of hydroxylapatite and its thermal decomposition products in the aggressive body environment calls for only sparingly soluble materials or hydroxylapatite-based composites with increased an *in vivo* resorption resistance. Hence, in addition to phosphate-based ceramics, many silicate-based ceramics are under scrutiny at present, owing to their known or expected ability to inhibit bone resorption and to promote osteoblast differentiation, osteocalcin secretion, and alkaline phosphatase activity. These ceramics include a variety of silicates such as sphene (titanite, calcium titanium silicate, CaTiOSiO_4_ [1,2]), the calcium silicates larnite (ß-Ca_2_SiO_4_ [3]) and wollastonite (α-Ca_3_[SiO_3_]_3_ [4]), the calcium magnesium silicates diopside (CaMgSi_2_O_6_ [5]), merwinite (Ca_3_MgSi_2_O_8_ [6]), monticellite (CaMgSiO_4_ [7]) and åkermanite (Ca_2_MgSi_2_O_7_ [8]), as well as the Zr-and Zn-sorosilicates baghdadite (Ca_3_Zr(O_2_[Si_2_O_7_])) [9] and hardystonite (Ca_2_Zn[Si_2_O_7_]) [10].

**Figure 1:**
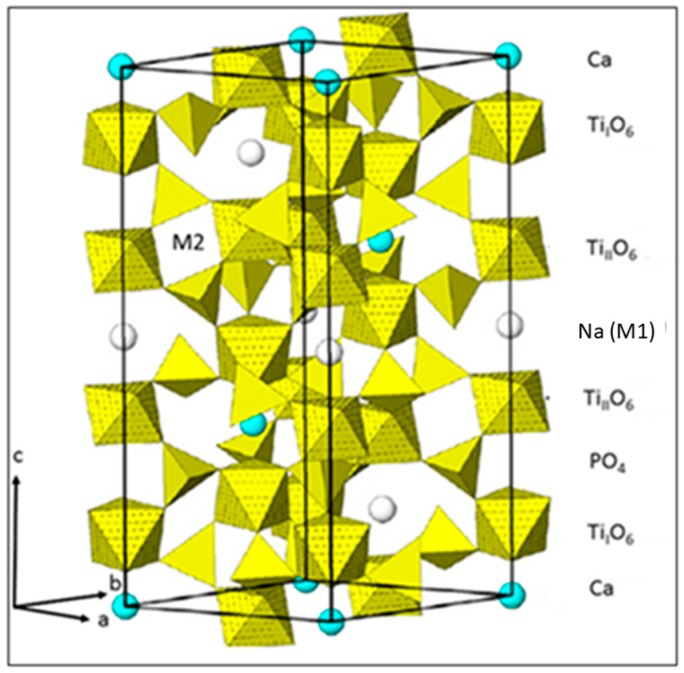
Unit cell of CaTi_4_(PO_4_)_6_ doped with Na ions located in M1 vacancies [12]. ©With the permission of the Mineralogical Society of America.

Other potentially useful materials include NaSiCon-type compositions with a [NZP] structure within the quaternary system CaO–P_2_O_5_–TiO_2_–ZrO_2_. NaSiCons (**Na s**uper**i**onic **con**ductors) obey the archetypical [NZP] (sodium zirconium phosphate) structure [11] with the rhombohedral space group $R\bar{3}c$ (167). Their composition can be described by the general formula **AM**_2_**X**_3_O_12_, where **A** = Ti, Zr, Hf, Nb or other transition metals of appropriate size, including Ln and Ac, **M** = Na, K, Ca or lattice vacancies, and **X** = P or Si. Figure 1 shows the unit cell of CaTi_4_(PO_4_)_6_. The Ti_2_(PO_4_)_3_^−^ groups are composed of a 3D-network of two TiO_6_ octahedra that are connected through their vertices to three PO_4_^3−^ tetrahedra. These basic units appear as –O_3_TiO_3_–O_3_TiO_3_– ribbons along the *c*-axis of the hexagonal unit cell. Within the *ab* plane, these ribbons are connected by PO_4_^3−^ tetrahedra.

The structural formula of NaSiCons is [M1][M2][A^VI^_2_][B^IV^_3_]O_l2_, where M1 and M2 are interstitial vacancy sites, either partially or fully occupied by appropriately sized cations. Small highly charged ions such as Zr or Ti occupy the octahedral A-sites, and Si or P fill the tetrahedral B-sites. Owing to the existence of lattice vacancies, an unusually large array of ions with widely varying oxidation states, Shannon ionic radii, and Pauling electronegativities can be accommodated. As shown in Figure 1, in the case of CaTi_4_(PO_4_)_6_ (CT_4_P_3_) (This nomenclature refers to the so-called cement-chemical notation as follows: C = CaO, T = TiO_2_, Z = ZrO_2_, P = P_2_O_5_. Hence, CT_4_P_3_ means CaO·4TiO_2_·3P_2_O_5_), the large M1 cavities with an octahedral geometry (Wyckoff position 6b in $R\bar{3}$c) along the *c* axis can be occupied by Na ions. The smaller M2 cavities with a trigonal-prismatic geometry (Wyckoff position 18e) located between the chains are normally empty and only filled if additional ion contributions are required for the charge compensation.

When the monovalent Na cation in synthetic [NZP] or the K cations in the natural mineral kosnarite (KZP) [13] are substituted by divalent cations such as Ca, Sr or Ba, the symmetry is lowered to $R\bar{3}$ (148). This happens by the ordering of cations and vacancies in the M2 cavities that results in the loss of the *c* glide plane owing to the fact that half the M1 sites are vacant [11]. As shown by Senbhagaraman et al. [14], the M1 site can be either completely empty as in the case of □Nb^5+^_2_(PO_4_)_3_, or partially filled as in □_0.5_Ca^2+^_0.5_Ti_2_(PO_4_)_3_ and □_0.66_Ln^3+^_0.33_Ti_2_(PO_4_)_3_. These vacancies □ account for the structural variability of the NaSiCon family, as well as their substantial ionic conductivity.

In this system, stoichiometric compounds exist with a much-improved mechanical and chemical stability compared to unsubstituted calcium orthophosphates. In addition, they have been shown to exhibit an enhanced osteoblastic differentiation, including a sufficient expression of a broad array of osteogenic markers, suggesting a high degree of osseoconductive potential. Consequently, these novel transition metal-substituted calcium hexaorthophosphates may find biomedical applications as long-term stable, sparingly soluble osseoconductive coatings for endoprosthetic implants and osseosynthetic fixation devices. When doped with small alkali ions with a high electron mobility such as lithium or sodium, they display a substantial solid-state ionic conductivity, suggesting their use as charge carriers in novel bioelectric systems including electroceuticals and bone growth-stimulating devices to treat nonunion (pseudoarthrosis) fractures.

Besides their potential biomedical applications, calcium (titanium, zirconium) hexaorthophosphates with a [NZP] (sodium zirconium phosphate) structure [11] have found other promising uses. All members of this family show low, sometimes even near-zero, coefficients of linear thermal expansion and, hence, a high thermal shock resistance [15] as well as a low solubility in aqueous solutions [16]. Moreover, these compounds display a substantial solid-state ionic conductivity, in particular when doped with small cations with a high electron mobility such as sodium or lithium, and display a high structural flexibility. The ionic conductivity may be utilized in anode materials for sodium-ion batteries [17], and the structural flexibility makes them amenable to use in radioactive waste management by allowing the incorporation of a very large array of cations in framework or inter-framework positions [18]. This behavior renders ceramics with a [NZP] structure a close to universal solid-state solvent for nuclear waste immobilization, with the potential to incorporate not only fission products such as radioactive Cs^+^ and Sr^2+^ ions but also a host of lanthanides (Ln) and actinides (Ac) [18,19]. Furthermore, [NZP] coatings have been suggested and explored as coatings for the high-temperature oxidation protection of carbon-carbon composites [20].

Since comparatively little attention has been paid to this novel class of bioceramics in the past, this contribution intends to raise awareness of the potential use of these ceramic materials in biomedicine by shedding light on their advantageous properties, which may stimulate additional research work and, eventually, clinical applications. This contribution summarizes the work of the author and his research group over the last two decades.

## 2. Materials and Methods

A powder synthesis was carried out by the sol-gel method, using calcium nitrate tetrahydrate, Ca(NO_3_)_2_·4H_2_O, titanium ethoxide, Ti(C_2_H_5_)_4_O_4_, zirconium tetrabutoxide, ZrCH_3_(CH_2_)_3_O_4_, and diammonium hydrogenphosphate, (NH_4_)_2_HPO_4_ [21]. The very fine-grained products were agglomerated by wet granulation, spray-dried to obtain plasma-sprayable powders, and classified by sieving into grain size ranges of + 45–71 µm (coarse) and + 25–45 µm (fine).

Commercially available Ti6Al4V substrates consisted of cylindrical rods (130 mm length, 12 mm diameter), grit-blasted with alumina particles (250 µm, 60 mesh) at a blasting angle of 75° and 4 bar air pressure at a distance of 100 mm. After grit-blasting, the specimens were cleaned ultrasonically in a 7.5% Tickopur^®^ solution (Bandelin electronic, Berlin, Germany) at 60 °C for 5 min, and air-dried.

### 2.1. Chemical Stability of Precursor Powders

**Figure 2:**
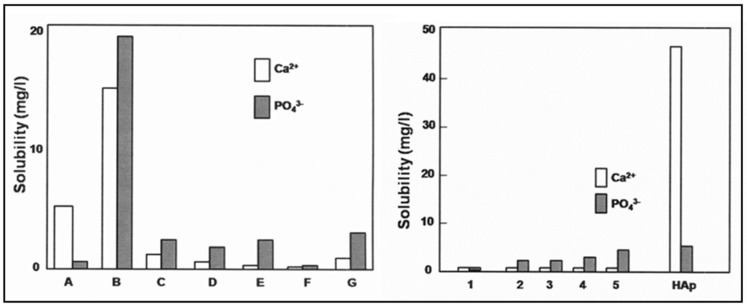
Left: Solubility of Ca(Ti,Zr) hexaorthophosphate powder compacts in 0.2 molar tris–hydroxymethyl–amino–methane–HCl (TRIS–HCl) at pH = 7.4 and 37 °C [16]. A Hydroxylapatite, B α–tricalcium phosphate, C CaTi_4_(PO_4_)_6_, D CaTi_3_Zr(PO_4_)_6_, E CaTi_2_Zr_2_(PO_4_)_6_, F CaTiZr_3_(PO_4_)_6_, G CaZr_4_(PO_4_)_6_. Right: Solubility of atmospheric plasma-sprayed CaTiZr_3_(PO_4_)_6_ coatings in 0.2 molar tris–hydroxymethyl–amino–methane–HCl (TRIS–HCl) at pH = 7.4 and 37 °C compared to hydroxylapatite coatings [16]. The numbers 1 to 5 refer to different sets of plasma-spray parameters, as specified in Table 1.

All members of the [NZP] family show a low solubility in aqueous solutions. This is of salient importance when bioceramic materials are required with a high resorption resistance in contact with extracellular fluid (ECF). The solubility of several CaTi_4-x_Zr_x_(PO_4_)_6_ compositions was tested on powder compacts in 0.2 molar tris–hydroxymethyl–amino–methane–HCl (TRIS-HCl) at a pH value of 7.4 and a physiological temperature of 37 ± 0.5 °C in a circulating reactor for up to 120 h [22]. The design and functioning of the reactor has been described in [23]. The minimum solubility in the TRIS–HCl buffer solution was obtained for CTZ_3_P_3_ powder compacts (composition F; see Figure 2, left). This composition was used in further studies.

### 2.2. Plasma Spraying of Powders

The coatings were deposited on Ti6Al4V substrates prepared, as described above, by conventional atmospheric plasma-spraying using an M1000 system (Plasma-Technik, Wohlen, Switzerland) in conjunction with a Sulzer Metco F4 plasmatron (Oerlikon Metco, Pfäffikon, Switzerland).

The powders with the composition CTZ_3_P_3_ were deposited according to a statistical experimental Plackett-Burman screening design [24], with 12 runs, where 11 independent factors were varied. Of those, only 7 were assigned, as shown in Table 1, and the remaining 4 unassigned factors were used to estimate the standard deviation of the coefficients of the response equations and, thus, the minimum factors significance. The response equations can be found elsewhere [25].

### 2.3. Coating Characterization

The microstructure and composition of the plasma-sprayed coatings were investigated by scanning electron microscopy (JSM-35C, JEOL Ltd., Akishima, Tokyo, Japan) and electron microprobe analysis (JX4 8900, JEOL Ltd., Akishima, Tokyo, Japan), respectively. Phase analyses were performed by X-ray diffraction (XRD7, Seifert-FPM GmbH, Freiberg, Germany) using CuK_α_ radiation at 35 kV and 20 mA with a scanning rate of 3° 2θ/min.

The coating porosity was determined according to the ASTM C373-88 designation [26], and the coating adhesion was measured by the ASTM C633-13 method [27]. The surface roughness data was obtained by a Mitutoyo 301 Surftest surface profilometer (Mitutoyo Europe GmbH, Neuss, Germany) equipped with a diamond stylus.

*In vitro* osseoblastesis tests were conducted using bone marrow cells (BMCs) derived from adult rats. The cells were incubated for 24 hours in 500 µl α-MEM + 15% fetal bovine serum + 10^−8^ mole dexamethason + 50 µg/ml ascorbic acid + 10 nmole ß–glycerophosphate, and seeded with a density of 3·10^4^ cells/cm^2^ onto the surface of CTZ_3_P_3_ disks sintered for 72 hours at 1300 °C. After a growth period of 2 weeks, the cells were fixed and stained with Giemsa stain (Biomol GmbH, Hamburg, Germany). Thermanox™ cell cover slips (ThermoFisher Scientificm Waltham, MA, USA) were used as control.

The *in vivo* tests involved implanting Ti6Al4V rods coated with a 150 µm thick plasma-sprayed layer of CTZ_3_P_3_ into the femoral medulla of skeletally mature sheep. After an observation period of 6 weeks, the sheep were sacrificed and the femora explanted and prepared. The procedures and techniques applied can be extracted from [28].

## 3. Results and Discussion

Table 2 summarizes the coating properties such as the solubility, porosity, adhesive strength, surface roughness, shear strength, and thickness, as functions of the parameter variation of the statistical experimental design that was used. The solubility, porosity and surface roughness are fairly constant, whereas the adhesive strength and shear strength vary widely, with thinner coatings revealing higher strength values. This large scatter indicates the need to further optimize the plasma-spray deposition conditions.

### 3.1. Solubility

Figure 2, left, shows the solubility of a series of Ca(Ti,Zr) hexaorthophosphates powder compacts compared to that of hydroxylapatite and α–tricalcium phosphate [16]. Sample F with the nominal composition of CTZ_3_P_3_ revealed a minimum solubility, as also confirmed by Ploska and Berger [29]. Consequently, plasma-sprayed coatings with this composition were used in further solubility tests, as shown in Figure 2, right.

### 3.2. Coating Microstructure

**Figure 3:**
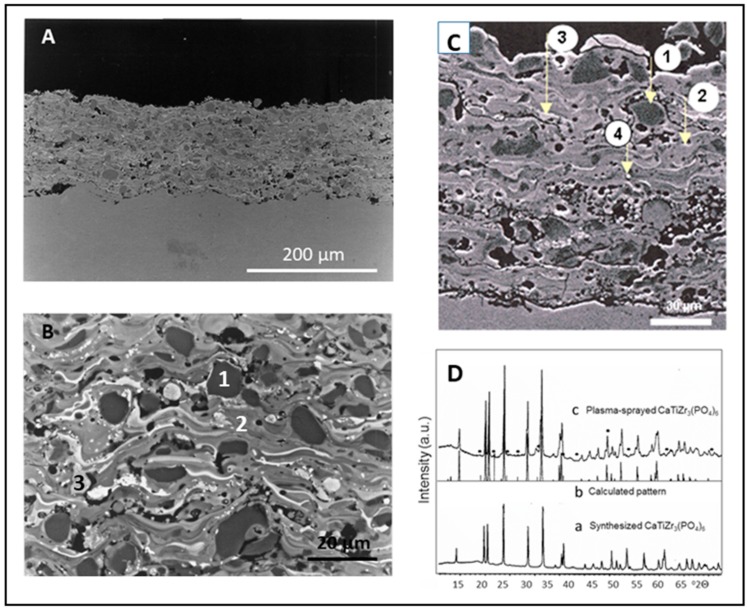
Cross-sectional images of plasma-sprayed CaTi_4-x_Zr_x_(PO_4_)_6_ coatings. (**A**): CZ_4_P_3_ coating (plasma power 36 kW, spray distance 90 mm, argon gas flow rate 50 slpm, hydrogen gas flow rate 10 slpm). (**B**): Enlarged image of (**A**) [30]. © Images courtesy of Dr. Guido Reisel, Oerlikon Metco. (**C**): CTZ_3_P_3_ coating (plasma power 35 kW, spray distance 120 mm, argon gas flow rate 50 slpm, hydrogen gas flow rate 12 slpm) [16,31]. (**D**): X-ray diffraction pattern of phase-pure CTZ_3_P_3_ starting powder (a), calculated interplanar spacings (b) and a plasma-sprayed coating (c). The dots indicate the presence of ZrP_2_O_7_, one of the decomposition products of incongruent melting.

The plasma-sprayed coatings revealed a pronounced lamellar and inhomogeneous microstructure, suggesting the heat-imposed alteration of the original homogeneous composition of the starting powder. Indeed, probing the composition by electron probe microanalysis (EPMA) confirmed the incongruent melting of the powder particles, which resulted in a mix of phosphorus-depleted calcium zirconium/titanium phosphates of various compositions. Figure 3 shows cross-sections of CZ_4_P_3_ coatings (A,B) and CTZr_3_P_3_ (C), as well as XRD charts of the starting powder (D, a), the calculated XRD pattern (D, b) and the integrated plasma-sprayed coating (D, c).

The coarse dark phase (1) shown in Figure 3B consists of the target phase CZ_4_P_3_, whereas the lighter appearing cellular striation phase (2) is depleted of ZrO_2_. The exsolved ZrO_2_ forms the white vermiform phase (3), delineating the streaky phase 2 as well as appearing as spherical deposits. Figure 3C shows a similar heat-imposed alteration of the target phase into at least four chemically different phases: Phase 1 (dark) consists of the CTZ_3_P_3_ target phase embedded in two lighter appearing, strongly phosphorus-depleted phases, with the approximate compositions Ca(Ti,Zr)_4.5_O_4.6_(PO_4_)_3.6_ (phase 2) and Ca(Ti,Zr)_4.2_O_6.85_(PO_4_)_1.7_ (phase 3). This suggests the reduction of PO_4_^3−^ ions by the comparatively high hydrogen content of the plasma gas (Table 1, column 3). Finally, the spherical phase 4 consisting of ß–ZrO_2_ (baddeleyite) attests to the complete decomposition of the target phase during the incongruent melting. Adjacent to the interphase between the coating and substrate, a noticeable porosity is apparent (see Table 2). Table 3 shows the compositions in mole% of the identified decomposition phases in comparison to that of the target CTZ_3_P_3_ phase.

### 3.3. Coating Porosity

The average porosity of plasma-sprayed Ca(Ti,Zr) hexaorthophosphate coatings was found to be 17 ± 4 vol% (N = 12; see Table 2). Whereas thin coatings (< 60 µm) showed a unimodal pore size distribution with a preferential average pore diameter of 11 µm, thicker coatings (> 180 µm) revealed a multimodal pore size distribution with frequency maxima of 12 and 36 µm.

### 3.4. Coating Adhesion

Theoretically, Ca(Ti,Zr) hexaorthophosphate coatings should adhere well to a Ti6Al4V substrate with a maximum adhesion strength exceeding 35 MPa [32], not the least owing to the generation of compressive coating stresses, the magnitude of which can be estimated using the following equation:

(1) $$\sigma_{c}=\left\{\frac{E_{c}\left(\alpha_{c}-\alpha_{s}\right)\Delta T}{1-\nu_{c}}\right\}+\left\{\frac{\left[\frac{1-\nu_{s}}{E_{s}}\right]d_{c}}{d_{s}}\right\}\;,$$

where α is the coefficient of the linear thermal expansion (CTE), ν is the Poisson ratio, E is the modulus of elasticity, and d is the thickness. The subscripts *c* and *s* refer to the coating and substrate, respectively [33]. The CTE of CTZ_3_P_3_ is anisotropic, with α[10.0] = 5 ppm and α[00.1] = 0.9 ppm [14]. The mean CTEs of different compositions were determined as follows: CTZ_3_P_3_: 1.14 ppm, CT_2_Z_2_P_3_: 4.3 ppm, and CZ_4_P_3_: 5.5 ppm [21].

Hence, α_c_ < α_s_ (α_s_ = α_Ti6Al4V_ = 8 ppm). Since the sign of the stress (tensile or compressive) depends on the sign of $\left(\alpha_{c}-\alpha_{s}\right)$, in this case compressive stress develops in the coating and will be compensated by tensile stress in the substrate adjacent to the interface. This compressive stress supports the enhanced coating adhesion, in contrast to hydroxylapatite coatings deposited by plasma-spraying onto the Ti6Al4V substrates. Here, α_c_ is, with about 11 ppm [34], slightly higher than α_s_, and consequently the sign of $\left(\alpha_{c}-\alpha_{s}\right)$ is positive, leading to tensile stresses within the coating that promote coating delaminations, as occasionally observed on plasma-sprayed hydroxylapatite coatings.

### 3.5. Mechanical Coating Properties

Plasma-sprayed CTZ_3_P_3_ coatings deposited on Ti6Al4V substrates exhibit several advantageous mechanical and chemical properties that suggest a successful performance in biomedical applications (Table 4).

A comparison of the properties of plasma-sprayed CTZ_3_P_3_ and hydroxylapatite (HAp) coatings shown in Table 4 indicates required property improvements of the former, in particular in terms of the adhesion and tensile strength, respectively as well as in terms of the shear strength. However, the substantially lower coefficient of the linear thermal expansion of CTZ_3_P_3_ coatings compared to HAp predicts the development of compressive coating stresses when applied to Ti6Al4V implant surfaces, as opposed to strong tensile stresses in the case of hydroxylapatite coatings. This makes a successful enhancement of the adhesive strength of CTZ_3_P_3_ coatings highly likely when the appropriate parameter optimization during plasma spraying will be carried out.

### 3.6. Electrical Properties

Among the advantageous properties of Ca(Ti,Zr) hexaorthophosphates are their solid ionic conductivity, in particular when doped with alkali ions with a high electron mobility such as lithium or sodium. However, a reliable quantitative data of the electric conductivity is far and apart in literature sources, and appear to be highly influenced by the chemical composition and thus, synthesis conditions, in particular the presence of unreacted starting compounds. The dielectric permittivity of Ca(Ti,Zr) hexaorthophosphates was reported to be 15.4, lower than that of densified hydroxylapatite, which showed values of around 20 at room temperature [37]. The ionic conductivity of Na- or Li-doped NaSiCon structures was reported to be in the range of 10^−2^ S∙m^−1^ [38], associated with the high concentration of charge-carrying alkali ions with a high electric mobility. This high electric mobility of alkali ions results from hopping among interstitial M1 and M2 sites (Figure 1), in contrast to less mobile Ca ions. Hence, to achieve a higher ionic conductivity of Ca(Ti,Zr) hexaorthophosphates, aliovalent doping with highly mobile Na or Li ions intercalated into the only partially occupied M1 sites of Ca(Ti,Zr) hexaorthophosphates appears to be a feasible route. Indeed, such doped compounds have been recently suggested as solid-state electrode materials for oxide-based fuel cells [39,40]. Based on their high ionic conductivity, alkali-doped Ca(Ti,Zr) hexaorthophosphates may be utilized in fourth-generation biomaterials [41], which allow integrating electronic systems with the human body to provide powerful diagnostic as well as therapeutic tools aimed toward basic research and clinical utilization. The functional advantages of such biomaterial systems include manipulating cellular bioelectric responses for tissue regeneration, as well as monitoring cellular responses directed at communication with host tissues via subtle bioelectric signals. Such novel systems could include bone growth-stimulating devices based on the equivalent circuit of a capacitor that, by appropriate poling, would store negative electrical charges close to the interface with the growing bone, thus enhancing the bone apposition rate as well as the bone density [12].

### 3.7. In Vitro Biocompatibility of Coatings

Among the earliest biomedical studies [42] on this novel class of bioceramics were *in vitro* biocompatibility tests that used L929 murine fibroblasts derived from normal subcutaneous areolar and adipose tissue of a 100-day old male C3H/An mouse, seeded onto a CZ_4_P_3_ powder substrate. The fibroblasts were found to be strongly attracted to the ceramic substrate, and the ceramic powder was phagocytized by the cells. No indication was found of the disturbance of either the cell adherence or phenotypical activity.

Subsequently, Knabe et al. [43] grew human bone-derived cells on sintered calcium (Ti,Zr) hexaorthophosphate samples with various compositions, and tested for the expression of a set of biochemical indicators for cell proliferation and cell vitality, including the upregulation of osteocalcin, osteonectin, osteopontin, alkaline phosphatase, and bone sialoprotein I. The compositions conforming specifically to CTZ_3_P_3_ displayed a maximum osteoblastic differentiation, including the sufficient expression of an array of osteogenic markers, thus suggesting a high degree of osseoconductive potential. Similar results were reported for the growth of bone-marrow stromal cells cultured on calcium titanium phosphate microspheres to be used as potential scaffolds for bone reconstruction [44].

### 3.8. Osteoblast Cell Culture Tests

*In vitro* biocompatibility tests with primary rat bone marrow cells (BMCs) showed a substantial cell proliferation in the presence of fetal bovine serum. Thermanox™ cell cover slips were used as control, against which the rating of the cell vitality and morphology was performed according to the classification of cells into four groups [45]: class I (areas without cells, isolated cells or dead cell detritus), class II (areas with loose reticular cell associations), class III (areas covered with a thick cell layer), and class IV (nodulary centers with a multi-layered cell formation) (Table 5).

Although no dramatic differences of the cell-biological behavior of the two substrates (Table 5) were observed, it can be concluded that 75% of the cells on sintered CTZ_3_P_3_ disks showed an improved cell growth (classes III + IV), as opposed to 66% on the control. Likewise, the percentage of missing, dead or deficient cells (1%) belonging to class I was noticeably lower on a sintered CTZ_3_P_3_ substrate compared to the control (7%).

### 3.9. Animal Model Tests

There has been some controversy related to the cytocompatibility of zirconia in contact with living tissue [46]. Histomorphological and morphometrical studies of the interface of glassy and ceramic biomaterials with a bony implantation bed [47] have shown that the presence of ZrO_2_ in biomaterials may be generally undesirable, as an incomplete transformation of chondroid and osteoid cells into osteoblasts was observed in a Sprague-Dawley rat femoral model, suggesting that zirconia-bearing ceramics could be counterproductive to bone bonding by inhibiting the matrix vesicle development and function [48,49].

However, *in vivo* biocompatibility tests conducted by Szmukler-Moncler et al. [42] with polycrystalline CZ_4_P_3_ disks implanted into the distal epiphyseal parts of the femora and tibiae of dogs have shown positive outcomes. After nine months of observation time, a direct and stable contact between the implant and bone had been established without an intervening pliable connective tissue capsule. Instead, an extensive remodeling of osteons had occurred, and neither the inflammation nor noticeable resorption of the biomaterial was observed. During the sample fracturing required for the specimen preparation, a separation occurred within the bulk bone matter but not along the bioceramic-bone interface, suggesting a strong and stable bone apposition. This early work was indeed a key experiment that established the *in vivo* osseoconductive capacity and stability of this novel bioceramic material.

**Figure 4:**
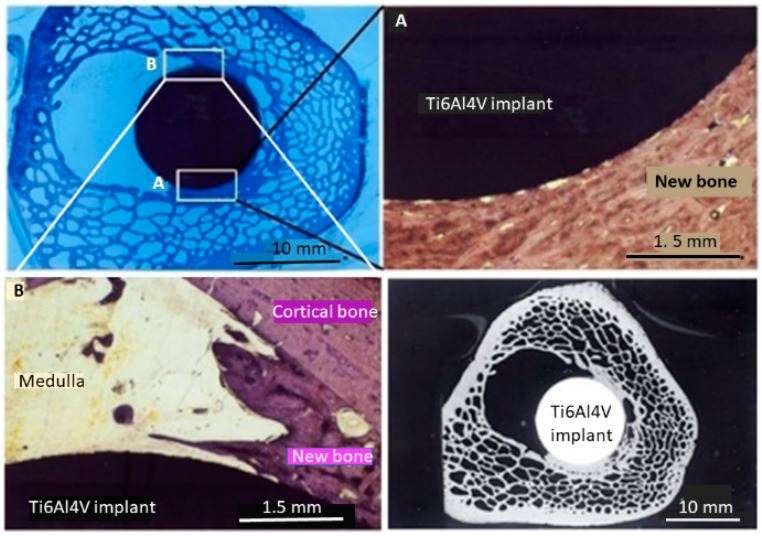
Formation of new bone in contact with a Ti6Al4V rod coated with a 150 µm thick plasma-sprayed layer of CTZ_3_P_3_ and implanted into the femoral medulla of sheep [25,28]. Top left: Micrograph of a distal section stained with toluidine blue. Top right: Magnified view of area A, showing the tight and continuous apposition of newly formed bone. Bottom left: Magnified view of area B, showing an incomplete gap-filling potential when the distance between the implant and cortical bone is too large. Bottom right: Cross-sectional microradiographic image of the implant located in the femoral medulla of sheep. © With permission by Wiley-VCH.

In addition, animal tests with a sheep model confirmed that a 150 µm thick CTZ_3_P_3_ coating applied to Ti6Al4V rods implanted in the femoral medulla led to the strong neoformation of dense bone at a stable interface implant-bioceramic coating without the coating delamination often observed with hydroxylapatite [28]. Figure 4, top left, shows a toluidine blue-stained distal section through the femoral medulla of sheep with an implanted coated Ti6Al4V rod. In Figure 4, top right, a magnified view of the area A shows the close integration of the implanted rod, characterized by a continuous and tight bone apposition via a thin lamella of newly formed bone. However, Figure 4, bottom left, reveals that in area B, showing the boundary toward the marrow-filled medullary cavity, the gap-bridging potential of the implant is reduced at larger distances between the coated implant and the surrounding cortical bone. Nevertheless, this preliminary study confirms, in an impressive way, the osteogenic potential of the novel class of bioceramic materials described in this contribution.

## 4. Conclusions and Implications

Bioceramic Ca(Ti,Zr) hexaorthophosphate coatings display advantageous biomedical properties, including a low solubility in simulated body fluid, a low to near-zero coefficient of thermal expansion, a high ionic conductivity, an adequate level of porosity, a reasonable adhesion to the Ti6Al4V implant surface, and a sufficient osteogenic potential. These properties suggest a successful application in biomedical contexts, ranging from osseoconductive coatings for the stem of hip endoprostheses and dental root implants, to osseosynthetic fixation devices and bioelectric systems. The latter may include bone-growth stimulators for the accelerated healing of operational traumata by speeding up bone growth, and may provide an important material-base for the treatment of osteoporotic conditions. This includes the potential use as a long-term stable bioceramic material in cases when, during an endoprosthetic replacement operation, the existing cortical bone bed had previously been damaged, in conjunction with and aggravated by an undesirable geometric bone configuration. In this case, the utilization of thin, rapidly dissolving standard hydroxylapatite-based coatings may not be the best choice to sustain a required large-scale and long-term bone regeneration. Even though in this situation much thicker hydroxylapatite coatings could be theoretically applied, such thick coatings are known to suffer from the build-up of substantial residual tensile stresses that would eventually result in the spalling, chipping or even complete delamination of the coating from the implant surface. Using thin (≤ 50 µm) coatings of materials with a much higher *in vivo* resorption resistance and the tendency to develop compressive coatings stresses owing to their low coefficient of thermal expansion could alleviate this problem. This is where Ca(Ti,Zr) hexaorthophosphates could potentially provide a powerful solution.

## Figures and Tables

**Table 1:** Plasma-spray parameter matrix chart according to a 12-run Plackett-Burman screening design [24] with 7 factors [21].

Run #	1	2	3	4	5	6	7
1	35	40	12	3	20/60	80	+45–71
2	35	50	6	6	20/60	80	+25–45
3	25	50	12	3	40/80	80	+25–45
4	35	40	12	6	20/60	120	+25–45
5	35	50	6	6	40/80	80	+45–71
6	35	50	12	3	40/80	120	+25–45
7	25	50	12	6	20/60	120	+71–45
8	25	40	12	6	40/80	80	+71–45
9	25	40	6	6	40/80	120	+45–25
10	35	40	6	3	40/80	120	+71–45
11	25	50	6	3	20/60	120	+71–45
12	25	40	6	3	20/60	80	+45–25

**1** plasma energy (kW), **2** argon gas flow rate (slpm), **3** hydrogen gas flow rate (slpm), **4** powder gas flow rate (slpm), **5** powder feed rate (% of maximum. First value: rotation of powder hopper screen, second value: rotation of stirrer), **6** spray distance (mm), **7** grain size range (µm). The run numbers 1 to 5 refer to the deposition conditions shown in Figure 2, right.

**Table 2:** Coating properties as functions of the parameter variation [21].

Run #	Solubility (mg/l)	Porosity (vol%)	Adhesive Strength (MPa)	Surface Roughness (µm)	Shear Strength (MPa)	Thickness (µm)
1	1.0	17	7.04	69.3	0.71	188
2	3.6	10	3.36	47.2	8.15	179
3	4.1	17	3.86	50.3	0.1	183
4	4.6	15	4.73	57.3	2.01	205
5	4.9	16	7.04	51.9	1,51	178
6	3.3	14	4.22	48.2	0.73	313
7	2.0	11	17.30	50.6	10.08	64
8	1.4	23	13.75	59.5	1.46	176
9	3.5	17	7.60	59.6	0.65	236
10	5.2	21	9.80	69.5	2.09	280
11	0.1	24	9.97	46.2	12.82	43
12	4.5	21	4.12	47.2	0.15	160

**Table 3:** Composition in mole% of the phases identified in Figure 3C.

Oxide	CaTiZr_3_(PO_4_)_6_	Phase 1	Phase 2	Phase 3	Phase 4
CaO	12.5	12.6	13.5	16.5	0
ZrO_2_	37.5	39.5	43.6	49.7	100
TiO_2_	12.5	12.9	18.0	19.8	0
P_2_O5	37.5	35.0	24.9	14.0	0

**Table 4:** Selected properties of plasma-sprayed CTZ_3_P_3_ coatings and performance requirements for commercial hydroxylapatite coatings [31,35]. See also [36].

Property	Dimension	CTZ_3_P_3_	HAp [32]	HAp [35]
Coating thickness range	µm	43–312	50–200	
Surface roughness R_a_	µm	46–70	>75	
Porosity	vol%	10–24		
Pore diameter	µm	10–40		
Adhesive strength (thickness > 200 µm)	MPa	≤17		
Adhesive strength (thickness < 100 µm)	MPa	≤32	>35	>51
Shear strength	MPa	≤13		>22
Coefficient of thermal expansion	ppm	1.14 [29]	10–14	
Solubility in pf-SBF	mg/l	<5		
Solubility in TRIS–HCl	mg/l	<1		

**Table 5:** Rating (%) of the cell vitality, proliferation and morphology.

Cell Class	Sintered CTZ_3_P_3_ Disks	Thermanox™ Control
I	1	7
II	24	27
III	27	24
IV	48	42

## References

[1] Biasetto L., Elsayed H., Bonollo F., Colombo P. (2016). Polymer-derived sphene biocoatings on cpTi substrates for orthopedic and dental implants. Surf. Coat. Technol..

[2] Elsayed H., Brunello G., Gardin C., Ferroni L., Badocco D., Pastore P., Sivolella S., Zavan B., Biasetto L. (2018). Bioactive sphene-based ceramic coatings on cpTi substrates for dental implants: An In Vitro study. Materials.

[3] Garcia E., Miranzo P., Sainz M.A. (2018). Thermally sprayed wollastonite and wollastonite-diopside compositions as new modulated bioactive coatings for metal implants. Ceram. Int..

[4] Buga C., Hunyadi M., Gácsi Z., Hegedüs C., Hakl J., Schmidt U., Ding S.J., Csik A. (2019). Calcium silicate layer on titanium fabricated by electrospray deposition. Mater. Sci. Eng. C.

[5] Sainz M.A., Pena P., Serena P., Caballero A. (2010). Influence of design on bioactivity of novel CaSiO_3_-CaMg(SiO_3_)_2_ bioceramics: In Vitro simulated body fluid test and thermodynamic simulation. Acta Biomater..

[6] Ardakani M.H., Moztarzadeh F., Rabiee M., Talebi A.R. (2011). Synthesis and characterization of nanocrystalline merwinite (Ca_3_Mg(SiO_4_)_2_) via sol-gel method. Ceram. Int..

[7] Kalantari E., Naghib S.M. (2019). A comparative study on biological properties of novel nanostructured monticellite-based composites with hydroxyapatite bioceramic. Mater. Sci. Eng. C.

[8] Diba M., Goudouri O.M., Tapia F., Boccaccini A.R. (2014). Magnesium-containing bioactive polycrystalline silicate-based ceramics and glass-ceramics for biomedical applications. Curr. Opin. Solid State Mater. Sci..

[9] Huang L., Ji H., Liang Y., Xie Y., Zheng X. (2011). Bone Replacing Material of Baghdadite Coated-Titanium Alloy, Useful for Inducing Formation of Bone-Like Apatite in Simulated Body Fluid, Comprises Titanium and Its Alloy as Matrix and Coating Is Deposited on Matrix by Plasma Spray Coating. Chinese Patent.

[10] Wu C.T., Chang J., Zhai W.Y. (2005). A novel hardystonite bioceramic: Preparation and characteristics. Ceram. Int..

[11] Alamo J. (1993). Chemistry and properties of solids with the [NZP] skeleton. Solid State Ion..

[12] Heimann R.B. (2017). Calcium (Ti, Zr) hexaorthophosphate bioceramics for electrically stimulated biomedical implant devices: A position paper. Am. Mineral..

[13] Brownfield M.E., Foord E.E., Sutley S.J., Botinelly T. (1993). Kosnarite, KZr_2_(PO_4_)_3_, a new mineral from Mount Mica and Black Mountain, Oxford County, Maine. Am. Mineral..

[14] Senbhagaraman S., Row G.T.N., Umarji A.M. (1993). Structural refinement using high-resolution powder X-ray diffraction data of Ca_0.5_Ti_2_P_3_O_12_, a low-thermal-expansion material. J. Mater. Chem..

[15] Alamo J., Roy R. (1986). Crystal chemistry of the NaZr_2_(PO_4_)_3_, NZP or CTP, structure family. J. Mater. Sci..

[16] Heimann R.B., Heimann R.B. (2012). Transition metal-substituted calcium orthophosphates with NaSiCon structure: A novel type of bioceramics. Calcium Phosphate. Structure, Synthesis, Properties, and Applications.

[17] Hung T.F., Lan W.H., Yeh Y.W., Chang W.S., Yang C.C., Lin J.C. (2016). Hydrothermal synthesis of sodium titanium phosphate nanoparticles as efficient anode materials for aqueous sodium-ion batteries. ACS Sustain. Chem. Eng..

[18] Scheetz B.E., Agrawal D.K., Breval E., Roy R. (1994). Sodium zirconium phosphate (NZP) as a host structure for nuclear waste immobilization: A review. Waste Manag..

[19] Vance E.R., Gregg D.J., Heimann R.B. (2012). Calcium phosphate materials for radioactive waste immobilization. Calcium Phosphate. Structure, Synthesis, Properties, and Applications.

[20] Agrawal D.K., Harshé G., Breval E., Roy R. (1996). [NZP], NaZr_2_P_3_O_12_-type materials for protection of carbon-carbon composites. J. Mater. Res..

[21] Heimann R.B., Schneider K. (2000). Final report DFG project, grant number He 923/9-1/2.

[22] Heimann R.B. (2006). In Vitro- und In Vivo-Verhalten von osteokonduktiven plasmagespritzten Ca-Ti-Zr-Phosphat-Beschichtungen auf Ti6Al4V-Substraten [In Vitro and In Vivo behavior of plasma-sprayed osseoconductive Ca-Ti-Zr phosphate coatings on Ti6Al4V substrates]. BIOmaterialien.

[23] Berger G., Gildenhaar R., Ploska U. (1995). Rapidly resorbable, glassy crystalline materials on the basis of calcium alkali orthophosphates. Biomaterials.

[24] Plackett R.L., Burman J.P. (1946). The design of optimum multifactorial experiments. Biometrika.

[25] Heimann R.B., Lehmann H.D., Heimann R.B., Lehmann H.D. (2015). Biological performance testing of bioceramic coatings. Bioceramic Coatings for Medical Implants. Trends and Techniques.

[26] ASTM C373-88 (2006). Standard Test Method for Water Absorption, Bulk Density, Apparent Porosity, and Apparent Specific Gravity of Fired Whiteware Products.

[27] ASTM C633-13 (2017). Standard Test Method for Adhesion or Cohesion Strength of Thermal Spray Coatings.

[28] Heimann R.B., Schürmann N., Müller R.T. (2004). In Vivo and In Vitro performance of Ti6Al4V implants with plasma-sprayed osteoconductive hydroxyapatite-bioinert bond coat ‘duplex’ systems: An experimental study in sheep. J. Mater. Sci. Mater. Med..

[29] Ploska U., Berger G. (1997). Solubility of compositions in the system CaTi_x_Zr_4-x_(PO_4_)_6_ with x = 0 − 4. Biomaterials.

[30] Reisel G. (1996). Entwicklung von HVOF- und APS-Gespritzten Biokeramischen Schichten für die Endoprothetik [Development of HVOF- and APS-Sprayed Bioceramic Coatings for Endoprosthetic Uses]. Masters Thesis.

[31] Schneider K., Heimann R.B., Berger G. (2001). Plasma-sprayed coatings in the system CaO-TiO_2_-ZrO_2_-P_2_O_5_ for long-term stable endoprostheses. Mater. Werkst..

[32] Wintermantel E., Ha S.W. (1996). Biokompatible Werkstoffe und Bauweisen. Implantate für Medizin und Umwelt [Biocompabible Materials and Constructions. Implants for Medicine and Environment].

[33] Heimann R.B. (2008). The third energy transfer process: Particle-substrate interaction. Plasma Spray Coating. Principles and Applications.

[34] Willmann G. (1999). Beschichtung von Implantaten mit Hydroxylapatite. Die Option auf eine stoffschlüssige Verbindung zwischen Knochen und Metall [Coating of implants with hydroxylapatite. The option of positive substance jointing of bone and metal]. Mater. Werkst..

[35] Callahan T.J., Gantenberg J.B., Sands B.E. (1994). Characterization and Performance of Calcium Phosphate Coatings for Implants.

[36] ASTM F1609–08 (2014). Standard Specification for Calcium Phosphate Coatings for Implantable Materials.

[37] Gittings J.P., Bowen C.R., Dent A.C.E., Turner I.G., Baxter F.R., Chaudhuri J.B. (2009). Electric characterization of hydroxyapatite-based bioceramics. Acta Biomater..

[38] Xie H., Li Y.T., Goodenough J.B. (2011). NaSiCon-type Li_1+2x_ Zr_2−x_ Ca_x_(PO_4_)_3_ with high ionic conductivity of room temperature. RSC Adv..

[39] Ortiz G.F., López M.C., Lavela P., Tirado J.L. (2014). Improved lithium-ion transport in NaSiCon-type lithium titanium phosphate by calcium and iron doping. Solid State Ion..

[40] Jolley A.G., Cohn G., Hitz G.T., Wachsman E.D. (2015). Improving the ionic conductivity of NaSiCon through aliovalent cation substitution of Na_3_Zr_2_Si_2_PO_12_. Ionics.

[41] Ning C.Y., Zhou L., Tan G.X. (2016). Fourth-generation biomedical materials. Mater. Today.

[42] Szmukler-Moncler S., Daculsi G., Delécrin J., Passuti N., Deudon C. (1992). Calcium-metallic phosphates: A new coating biomaterial?. Adv. Biomater..

[43] Knabe C., Berger G., Gildenhaar R., Klar F., Zreiqat H. (2004). The modulation of osteogenesis In Vitro by calcium titanium phosphate coatings. Biomaterials.

[44] Barrias C.C., Ribeiro C.C., Lamghari M., Miranda C.S., Barbosa M.A. (2005). Proliferation, activity, and osteogenic differentiation of bone marrow stromal cells cultured on calcium titanium phosphate microspheres. J. Biomed. Mater. Res. A.

[45] Maniatopoulos C. (1988). Osteoblast cell cultures: A testing model for biomaterials. Cell Tissue Res..

[46] Bernstein A., Nöbel D., Mayr H., Göbel T., Berger G., Ploska U., Gildenhaar R., Brandt J. (2008). Inhibition of mineralization by a calcium zirconium phosphate coating. J. Biomed. Mater. Res. Part B Appl. Biomater..

[47] Gross U., Strunz V. (1985). The interface of various glasses and glass ceramics with a bony implantation bed. J. Biomed. Mater. Res..

[48] Gross U., Müller-Mai C., Berger G., Ploska U. (2003). The tissue response to a novel calcium zirconium phosphate ceramics. Key Eng. Mater..

[49] Gross U., Müller-Mai C., Berger G., Ploska U. (2004). Do calcium zirconium phosphate ceramics inhibit mineralization?. Key Eng. Mater..

